# SnSe Nanosheets Mimic Lactate Dehydrogenase to Reverse Tumor Acid Microenvironment Metabolism for Enhancement of Tumor Therapy

**DOI:** 10.3390/molecules27238552

**Published:** 2022-12-05

**Authors:** Heng Wang, Beilei Wang, Jie Jiang, Yi Wu, Anning Song, Xiaoyu Wang, Chenlu Yao, Huaxing Dai, Jialu Xu, Yue Zhang, Qingle Ma, Fang Xu, Ruibin Li, Chao Wang

**Affiliations:** 1 Institute of Functional Nano & Soft Materials (FUNSOM), Soochow University, Suzhou 215123, China; 2 State Key Laboratory of Radiation Medicine and Protection, School for Radiological and Interdisciplinary Sciences (RAD-X), Collaborative Innovation Center of Radiation Medicine of Jiangsu Higher Education Institutions, Soochow University, Suzhou 215123, China

**Keywords:** lactate, nanozyme, tumor acidity, tumor microenvironment

## Abstract

The acidic tumor microenvironment (TME) is unfriendly to the activity and function of immune cells in the TME. Here, we report inorganic nanozymes (i.e., SnSe NSs) that mimic the catalytic activity of lactate dehydrogenase to degrade lactate to pyruvate, contributing to the metabolic treatment of tumors. As found in this study, SnSe NSs successfully decreased lactate levels in cells and tumors, as well as reduced tumor acidity. This is associated with activation of the immune response of T cells, thus alleviating the immunosuppressive environment of the TME. More importantly, the nanozyme successfully inhibited tumor growth in mutilate mouse tumor models. Thus, SnSe NSs show a promising result in lactate depletion and tumor suppression, which exemplifies its potential strategy in targeting lactate for metabolic therapy.

## 1. Introduction

The tumor microenvironment (TME) is a complex, heterogeneous ecosystem that supports tumor growth and promotes immune evasion [1]. A low pH, oxygen level and nutrient availability can all prevent antitumor immunity and effector cell functions [2,3]. Therefore, reversing the immunosuppressive TME and restoring the antitumor immune function of the immune system is an effective strategy to enhance tumor therapy [4]. An acidic environment is one of the characteristics that distinguishes tumors from other normal tissues [5]. Normal cells maintain their life activities through mitochondrial respiration, called oxidative phosphorylation (OXPHOS) [6]. Cancer cells primarily use glycolysis to rapidly obtain energy for metabolism, even in the presence of normoxia [7]. This is a distinctive feature of the metabolism of the tumor microenvironment and is known as the “Warburg effect”, or aerobic glycolysis [8]. Lactate, a major component of the TME produced by Warburg metabolism, is an important regulatory factor of the acidic TME [9,10]. Some immune cells in the TME, such as T cells, dendritic cells (DCs), and macrophages, sense high levels of extracellular lactate levels via MCT1 and MCT2, which trigger intracellular signals that affect their function to a dysfunctional status [9,11,12,13].

Lactate dehydrogenase is a glycolytic enzyme that catalyzes the dehydrogenation of lactate to pyruvate and plays an important role in the lactate metabolic pathway [14]. A nanozyme called two-dimensional (2D) SnSe nanosheets (NSs) was recently found, which mimics the catalytic activity of natural dehydrogenases in cellular metabolism and efficiently catalyzes the hydrogen transfer of the 1-(R)-2-(R′)-ethanol moiety, thereby converting lactate to pyruvate [15]. Factors such as size, structure, and environmental pH of inorganic nanomaterials can affect their enzymatic-like activity [16]. Compared to natural dehydrogenases, such a structure maintains good catalytic activity in most environments [17,18]. The dehydrogenase-like activity of SnSe NSs can be used not only to deplete tumor lactate, but also to elevate tumor pH and neutralize TME acidity, thus reversing the acidic TME.

Here, the synthesized SnSe NSs adsorb lactate onto its surface to produce an adsorption structure, and the two hydrogen atoms on the lactate are transferred to the Sn and Se atoms and separated as pyruvate [15]. In this way, we found that lactate dehydrogenase action simulated by SnSe NSs showed good lactate depletion levels both in vitro and in vivo. In addition, SnSe NSs-mediated lactate depletion reversed the suppression of immune cell function in the lactate-induced TME and inhibited tumor growth in multiple mouse tumor models. Taken together, SnSe NSs can effectively improve the acidic environment by depleting lactate in tumors, thus reversing the TME of immunosuppression. This study is expected to provide new ideas for targeted tumor metabolic therapy.

## 2. Results

### 2.1. Characterization and Functional Evaluation of SnSe NSs

**Figure 1:**
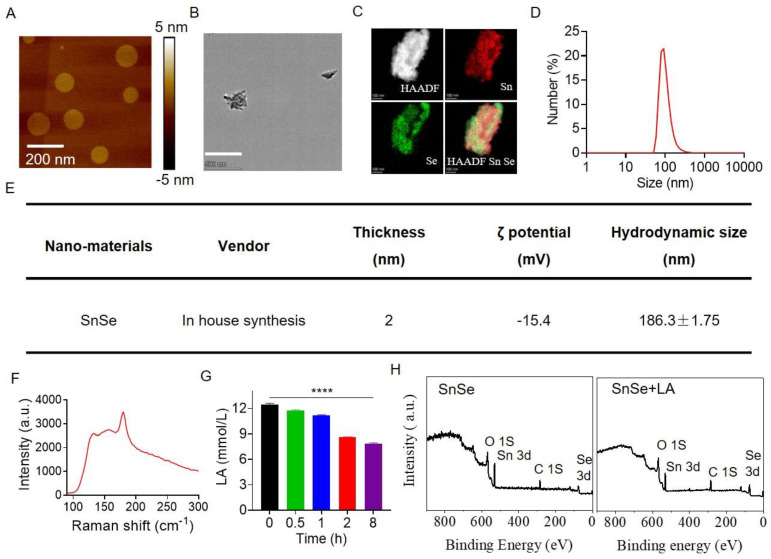
Characterization and functional verification of SnSe NSs. (**A**) AFM image of SnSe NSs. (**B**) TEM images of SnSe NSs. Scale bar: 500 nm. (**C**) Elemental mapping of SnSe NSs. (**D**) The primary size of SnSe NSs measured by Nano Measurer. (**E**) Table showing the material characteristics analyses of SnSe NSs. (**F**) Depicts the Raman shift of the SnSe NSs. (**G**) Evaluation of lactate consumption at different time points after incubation with SnSe NSs. (**H**) Whole XPS patterns of SnSe NSs and SnSe NSs incubated with lactate. Data are means ± SDs. Statistical significance was calculated using one-way ANOVA followed by Tukey’s post-test. *p*-value: **** *p* < 0.0001. a.u., arbitrary units.

Layered SnSe is an emerging black phosphorus structure [19]. Owing to the increase in the number of layers, the catalytic activity of SnSe nanosheets decreases significantly [15]. As shown in Figure 1A,E, we prepared single-atom layer SnSe nanosheets with a thickness of 2 nm. Atomic force microscopy showed that the primary size of single laminar SnSe nanosheets was approximately 120 nm (Figure 1A). The prepared SnSe NSs have an irregular planar structure (Figure 1B). The elemental mapping results show that Sn and Se constitute the nanomaterials (Figure 1C). The size of SnSe NSs was calculated statistically by a Nano Measurer as 120.5 ± 4.56 nm, which was similar to the AFM result (Figure 1D). The hydrodynamic size and zeta potential of SnSe NSs were measured as 186.3 ± 1.75 nm and −15.4 mV, respectively (Figure 1E). Raman spectroscopy is an important tool for researching the thickness and crystallinity of two-dimensional materials [20]. The Raman spectrum of SnSe NSs was obtained in the range of 100–400 (cm^−1^) using laser excitation at 514 nm. The Raman shift was observed at 179 (cm^−1^) (Figure 1F). Relatively weak intensity Raman shifts can also be found in the Raman spectra at 133 and 156 (cm^−1^), as shown in Figure 1F, which may be attributed to the near surface part of SnSe NSs. To evaluate the lactate consumption capacity of the nanomaterials, SnSe NSs were added into lactate solution (10 mM). The concentration of lactate in the mixed solution with the addition to SnSe NSs decreased with time (Figure 1G), indicating that the dehydrogenase catalytic activity of SnSe NSs was maintained under physiological conditions. To further understand the biochemical transformation of SnSe NSs and their driving metabolic mechanisms, we compared the metabolic transformation profiles of SnSe NSs in the presence and absence of lactate solution. XPS was used to observe the structural changes of SnSe NSs upon reaction with lactate. With the absence of lactate, there were obvious Sn and Se peaks in the full spectrum. However, after the reaction with lactate, the elemental Sn peaks appeared to decrease, indicating that Sn was metabolized by lactate and reacted better with lactate than Se (Figure 1H). Together, these results indicated that SnSe NSs were successfully prepared as mimetic lactate dehydrogenase nanozymes with powerful catalytic capacity.

### 2.2. Analyses of Lactate Consumption Behaviors of SnSe NSs

**Figure 2:**
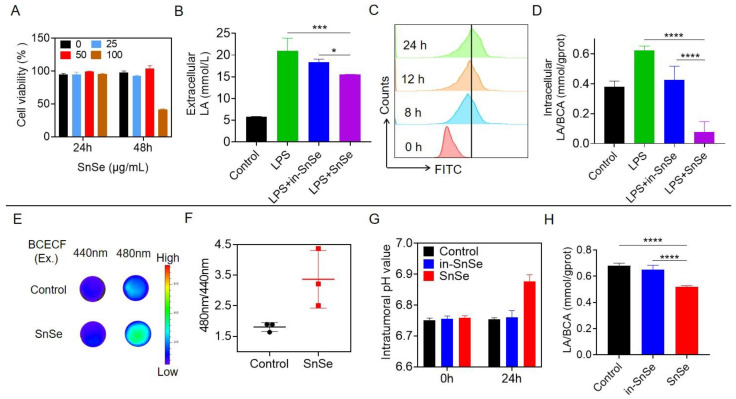
Analyses of lactate consumption behaviors of SnSe NSs. (**A**) Cell viability of RAW 264.7 cells after treatment with various concentrations of SnSe NSs. (**B**) RAW 264.7-cell extracellular lactate levels under LPS treatments with the addition of SnSe NSs and in-SnSe NSs for 24 h. (**C**) Flow cytometric assay of cell uptake of SnSe NSs at different time points. (**D**) The evaluation of intracellular lactate levels in RAW 264.7 cells in the presence of LPS treatments after incubation with SnSe NSs and in-SnSe NSs for 24 h. (**E**) In vivo fluorescence imaging of tumor tissue supernatant injected with SnSe NSs after 24 h. (**F**) Semiquantitative analysis of pH fluorescence intensities based on the imaging data shown in (E). (**G**) Intratumoral pH value of tumors at 0 and 24 h post-intravenous injection of SnSe NSs and in-SnSe NSs. (**H**) The lactate consumption level after different treatments in vivo. Data are means ± SDs. Statistical significance was calculated using one-way ANOVA followed by Tukey’s post-test. *p*-value: * *p* < 0.05; *** *p* < 0.001; **** *p* < 0.0001. a.u., arbitrary units.

SnSe NSs act as nanocatalysts for extra/intracellular lactate treatment with lactate depletion and neutralization of tumor acidity. First, to investigate the effect of SnSe NSs on cytotoxicity, different concentrations of samples were incubated with macrophages (RAW 264.7) for 24 h and 48 h. The results showed that there was no significant cytotoxicity of SnSe NSs concerning cells at various concentration ranges for 24 h, However, a high concentration of SnSe NSs (100 μg/mL) had a cytotoxic effect on macrophages for 48 h (Figure 2A), probably due to the fact that more NSs were swallowed by macrophages. Next, we also test the effect of SnSe NSs on cytotoxicity in cancer cells (B16-Luc, 4T1-Luc and CT26-Luc). The results showed that SnSe NSs had no significant cytotoxicity in cancer cells within a certain concentration range (Figure S1). Subsequently, we evaluated the lactate consumption capacity of SnSe NSs at the intracellular and extracellular levels. Inactive SnSe NSs were used as a control. Lipopolysaccharide (LPS)-stimulated macrophages have a Warburg metabolism similar to that of tumor cells, characterized mainly by glucose consumption and increased lactate amounts [21]. SnSe NSs and inactive SnSe NSs were treated with LPS-stimulated macrophages. Compared with SnSe NSs without dehydrogenase activity (in-SnSe), the lactate concentration in the supernatant of extracellular macrophages was decreased after SnSe NSs treatment (Figure 2B). Furthermore, we investigated the uptake of SnSe NSs with macrophages at different time points. Confocal fluorescence imaging and flow cytometry analysis showed that, with increasing time, FITC-labelled SnSe NSs were efficiently taken up by macrophages (Figure 2C and Figure S2). The lowest intracellular lactate levels were also detected in macrophages treated with SnSe NSs among the control groups. These results suggested the lactate depletion ability of SnSe NSs (Figure 2D).

Acidification of the extracellular environment was enhanced by an increase in glucose metabolism of lactate in tumor cells [22]. We hypothesized that SnSe NSs could react with lactate in tumors to neutralize tumor acidosis, which is one of the main markers of the TME [5]. To further investigate whether SnSe NSs can indeed deplete lactate in tumors, we next tested their effect in vivo. We established a subcutaneous CT26 tumor model. When the CT26 tumors grew up to 200–300 mm^3^, the subcutaneous CT26 murine tumors were left untreated or intratumorally injected with SnSe NSs at a dose of 4.5 mg/kg. We confirmed the ability of SnSe NSs to modulate tumor acidity by the pH-sensitive fluorescent dye 2,7-biscarboxyethyl-5(6)-carboxyfluorescein (BCECF) with ratio metric fluorescence imaging (Figure 2E,F). Then, the pH values within the tumors of SnSe NSs-treated mice were monitored by invasive pH microelectrodes. Remarkably, intratumoral injection of SnSe NSs neutralized the acidic tumor pH at 24 h post-injection, whereas in-SnSe NS injection had little effect on intratumoral pH (Figure 2G). In addition, the intratumoral lactate levels were measured by a lactate assay kit. The SnSe NSs showed the lowest relative level of lactate in tumors, while the lactate level of in-SnSe NSs was basically unchanged compared to PBS injection (Figure 2H). In conclusion, SnSe NSs exhibited more effective tumor lactate depletion and tumor acid neutralization than controls, which was attributed to their role as well functioning mimetic lactate dehydrogenase activity and tumor acidity regulators.

### 2.3. Evaluation of the Antitumor Efficacy of SnSe NSs 

**Figure 3:**
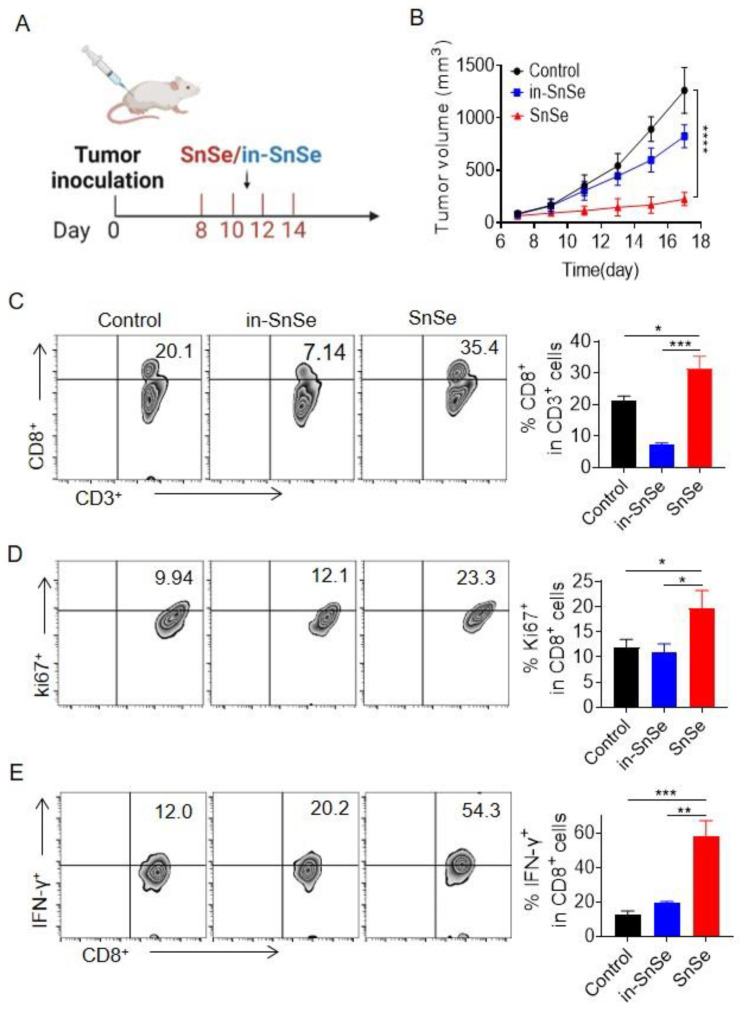
Evaluation of the antitumor efficacy of SnSe NSs. (**A**) The treatment scheme of SnSe NSs or in-SnSe NSs. (**B**) The tumor growth curve in CT26 subcutaneous tumor mice with various treatments (*n* = 5). (**C**–**E**) Representative flow cytometry zebra plots and statistical analyses of immune cells in mice with different treatments. (**C**) CD8^+^ T cells. (**D**) Proportion of Ki67 within CD8^+^ T cells. (**E**) Quantitative measurement of IFN-γ^+^ within CD8^+^ T cells. Data are means ± SDs. Statistical significance was calculated using one-way ANOVA followed by Tukey’s post-test. *p*-value: * *p* < 0.05; ** *p* < 0.01; *** *p* < 0.001; **** *p* < 0.0001. a.u., arbitrary units.

The antitumor effects of SnSe NSs were evaluated in a subcutaneous CT26 tumor model. According to the treatment protocol (Figure 3A), we treated tumors in mice with four intratumoral injections of SnSe NSs. Inactive SnSe NSs were used as a control. The mice receiving SnSe NSs showed a significant effective tumor inhibition effect, while the in-SnSe NSs treatment had a low suppression effect on tumors (Figure 3B). It is possible that this is due to in-SnSe NSs as foreign nanomaterials that are recognized by the immune system and which may promote the activity of immune cells [16]. It may be beneficial to improve the anti-tumor effect by promoting local inflammation. Due to its effective tumor lactate depletion and tumor suppression, we then further analysed TME cellular components, particularly T cells at day 18. The CD8^+^ T cells were promoted, indicating activation of T cell-mediated anti-tumor immune capacity (Figure 3C). T cells are an important component of the adaptive immune response [23]. Thus, the results indicated that SnSe NS-treated mice are able to induce relatively strong immune responses against tumors as compared to in-SnSe NS treatment. The percentage of Ki67 in CD8^+^ T cells was approximately one-fold higher in SnSe NS-treated tumor than in control and in-SnSe NS-treated tumors (Figure 3D), which suggested that T cell proliferation was promoted. The percentage of interferon-γ (IFN-γ) in CD8+ T cells was significantly increased after SnSe NS treatment compared to in-SnSe NSs and untreated mice, confirming the activation of T cell immune function in vivo by the nanoenzyme (Figure 3E). In summary, intratumor injection of SnSe NSs is able to activate immune cells, resulting in effective anti-tumor immunity.

### 2.4. SnSe NSs Promote Antitumor Efficacy in Multiple Tumor Models

**Figure 4:**
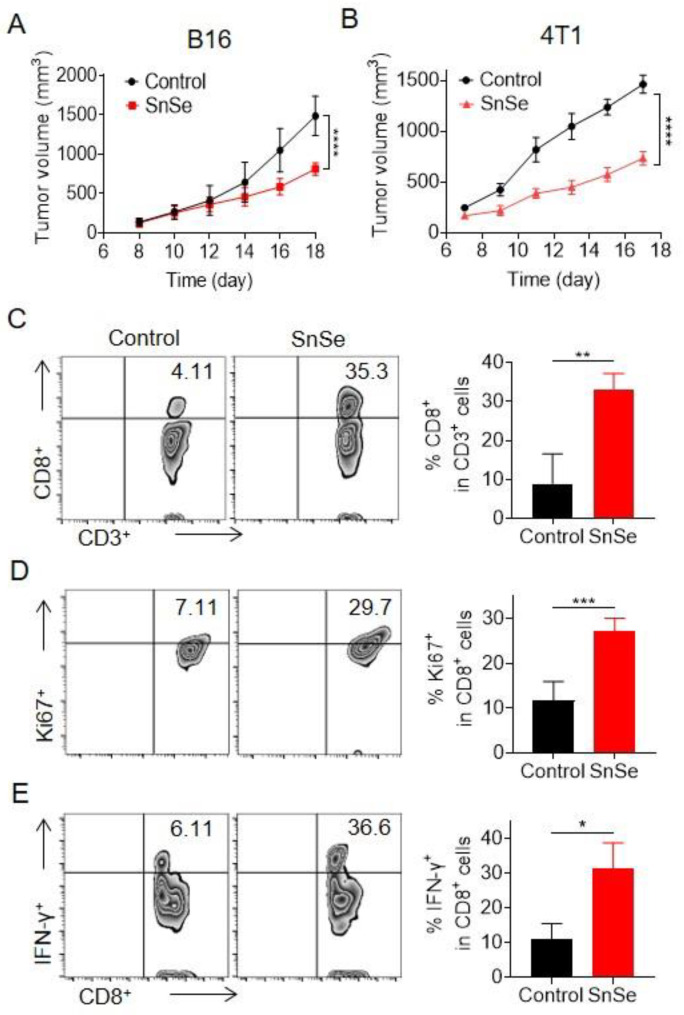
Antitumour therapy of SnSe NSs in multiple tumor models. (**A**,**B**) The tumor growth curves in B16 and 4T1 subcutaneous tumor mice treated with SnSe NSs (*n* = 5). (**C**–**E**) Representative flow cytometry zebra plots and statistical analyses of immune cells in the B16 tumor model with SnSe NS treatments. (**C**) CD8^+^ T cells. (**D**–**E**) Quantitative analysis of Ki67 and IFN-γ within CD8^+^ T cells. Data are means ± SDs. Statistical significance between different groups was obtained by Student’s *t*-tests (two-tailed). *p*-value: *p < 0.05; ** *p* < 0.01; *** *p* < 0.001; **** *p* < 0.0001. a.u., arbitrary units.

To extend SnSe NSs in different tumor models, we established a B16 melanoma model, as well as a 4T1 breast cancer model, by subcutaneous injection, where B16 melanoma cells and 4T1 breast cancer cells can form more aggressive and weakly immunogenic tumors. Mice bearing B16 or 4T1 were treated with SnSe NSs individually starting on day eight. After 14 d of administration (four intratumoral injections in total), subcutaneous tumors developed rapidly when the mice were left untreated. However, SnSe NSs also significantly inhibited the size of B16 and 4T1 tumors, demonstrating their broad spectrum antitumour effect in a variety of tumor models (Figure 4A,B). We verified the alteration of immune cells in the tumor microenvironment after administration by flow cytometry analysis. Similar to our previous data, in the B16 tumor model, the proportion of CD8^+^ T and CD4^+^ T cells in the SnSe NS-treated group was increased (Figure 4C and Figure S3). Moreover, the proportions of Ki67^+^, and IFN-γ^+^ CD8^+^ T cells were all increased (Figure 4D,E), confirming the proliferative and killing effects of T cells in vivo. In total, SnSe NS treatment can significantly activate the immune response in different tumor models to promote antitumour efficacy.

## 3. Materials and Methods 

### 3.1. Materials Synthesis

The SnSe nanosheets were prepared according to a previously reported method with a little modification [24]. One gram of SnSe material was weighed and mixed with 64 mL of 2% Pluronic F68 solution. After 8 h of ultrasonic shaking separation in an ice bath, it was mixed with 12 mL of 60% iododiol and centrifuged at 8000 rpm for 10 min at 22 °C to obtain the supernatant and pellet. The supernatant was then centrifuged at 20,000 rpm to obtain microsphere pellets and a brownish red supernatant. The brownish red supernatant was then centrifuged at 50,000 rpm to obtain another microsphere pellet. Alternatively, the pellet after the first centrifugation was lyophilized and resuspended in 32 mL of 2% PF-68 by sonication for 4 h. The resulting solution was mixed with 6 mL of 60% iododiol and centrifuged at 20,000 rpm to obtain a black supernatant, followed by two centrifugations at 50,000 and 80,000 rpm to obtain microsphere pellets [15]. All the latter microsphere pellets were washed three times with DI water to obtain SnSe nanosheets, while inactive SnSe nanosheets were obtained by mixing the SnSe materials with DI water and leaving it for a long time.

### 3.2. Characterization of Materials

The structural morphologies and thicknesses of SnSe NSs were measured by AFM (Dimension Icon, Bruker, USA) using ScanAsyst mode. The micromorphology and element distribution of SnSe NSs were used for characterization by field emission transmission electron microscope TALOS 200X (Thermo Fisher Scientific, Waltham, MA, USA). The primary size of the SnSe NSs was calculated and analysed by Nano Measurer statistics. The dynamic light scattering (DLS) measurement and zeta potential of SnSe NSs were determined using a Zetasizer nano ZS instrument (Malvern, Malvern, UK). Raman analysis was performed at room temperature by laser Raman spectroscopy (Bruker Optics, Tensor 27, Borken, Germany). 

### 3.3. Enzymatic Activity Assay

To evaluate the LDH activities of SnSe NSs, the reactants and substrates were mixed with SnSe NSs at 60 μg/mL and incubated with shaking at 37 °C. The reaction suspension was centrifuged, and 100 μL of the supernatant was collected at 0.5, 1, 2, and 8 h. A lactate assay kit was used to detect the absorbance of the supernatant at 530 nm on a microplate reader to measure lactate consumption. The changes in the chemical composition of SnSe NSs after the addition of lactate were examined by X-ray photoelectron spectroscopy (XPS) (Escalab 250Xi, Thermo Fisher, Waltham, MA, USA).

### 3.4. Cell Lines

All cell lines were purchased from the Cell Bank of Shanghai Institute of Biological Sciences, Chinese Academy of Sciences. B16F10-luc and RAW 264.7 cells were cultured in Dulbecco’s modified Eagle’s medium (DMEM), containing 10% fetal bovine serum (FBS; Gibco) and 1% penicillin–streptomycin. CT26- Luc and 4T1-Luc cells were cultured in Roswell Park Memorial Institute (RPMI) 1640 media containing 10% fetal bovine serum (FBS; Gibco) and 1% penicillin–streptomycin. All cells were incubated in a 5% CO_2_-containing atmosphere at 37 °C [25].

### 3.5. Mice 

BALB/c mice (6–8 weeks, female) and C57BL/6 mice (6–8 weeks, female) were obtained from Changzhou Cavens Experimental Animal Co. Ltd. After the sizes of the experimental groups were defined to balance statistical power, feasibility, and ethical aspects, they were approved by the regulatory authorities for animal welfare [26]. For mice experiments, the number of animals used in each group was 5. All our mouse experiments were performed in accordance with the animal protocol approved by the Laboratory Animal Center of our university.

### 3.6. In Vitro Cytotoxicity Assessment

RAW 264.7, B16, 4T1, and CT26 cells were inoculated into 96-well plates at a density of 5000 cells/well and cultured for 24 h. Then, RAW 264.7 cells were treated with different concentrations of SnSe NSs and cultured for 24 h and 48 h. Cancer cells were treated with different concentrations of SnSe NSs and cultured for 48 h. The CCK8 assay kit was used to detect the effect of SnSe NSs on the toxicity of RAW 264.7, B16, 4T1, and CT26 cells. 

### 3.7. Lactate Treatment of SnSe NSs in Macrophages

RAW 264.7 cells were inoculated into 24-well plates at a density of 100,000 cells/well. After cell adherence, LPS (40 ng/mL), SnSe NSs (60 μg/mL), or in-SnSe NSs (60 μg/mL) were added and incubated for 24 h. After 24h, the cells were aspirated and collected from each well plate. Then, the samples were centrifuged, and the supernatant was collected. The extracellular lactate consumption of cells was detected in the absorbance of the supernatant at 530 nm on a microplate reader ((Molecular Devices, Silicon Valley, CA, USA) with a lactate assay kit. Next, 1 mL of cell lysate was added to the cells obtained after centrifugation to split them for 1–2 h, and then they were ultrasounded every 20 min for 5 min during this process. Finally, the samples were centrifuged, and the supernatant was collected. The intracellular lactate consumption of RAW 264.7 cells was also detected in the absorbance of the final supernatant by the lactate assay kit. The BCA Protein Assay Kit (Beyotime Biotechnology) was used to determine the protein concentration of the supernatant to perform normalized data processing. The intracellular lactate level was calculated using the formula: the absorbance/protein concentration.

### 3.8. In Vitro Cellular Uptake Experiment

To assess the ability of cells to take up SnSe NSs, RAW264.7 cells were incubated with FITC-labelled SnSe NSs at different time points. In flow cytometry experiments, cells were collected to detect the ability of FITC-labelled SnSe NSs to be phagocytosed by macrophages using flow cytometry. In confocal experiments, cells were washed 3 times with PBS and then fixed with 4% paraformaldehyde for 30 min. Next, nuclei were stained with DAPI (4′,6-diamino-2-styrenol) for 10 min. The phagocytic ability of macrophages was observed by confocal microscopy (Zeiss LSM 800, Jena, Germany).

### 3.9. Tumor pH Treatment of SnSe NSs 

To study the tumor acidity neutralization capacity of SnSe NSs, intratumoral pH values of CT26 tumorbearing mice before and at 24 h after intratumoral injection of SnSe NSs and inSnSe NSs (*n* = 3 per group, SnSe or inSnSe NSs = 4.5 mg/kg) were measured using an invasive pH microelectrode (PMHP5, PreSens, Regensburg, Germany). In addition, excised tumors with 1 mL PBS from different groups were prepared in tumor homogenate by a tissue homogenizer. The tumor homogenates untreated and treated with SnSe NSs incubated in buffers at a pH value of 6.5 were then mixed with the pH responsive fluorescence dye BCECF (10 μM, ApexBio) for 10 min before being imaged under a Lumina III in vivo imaging system (PerkinElmer, Waltham, MA, USA) (excitation = 440 and 480 nm, emission = 520 nm).

### 3.10. Evaluation of Lactate Consumption In Vivo

When the subcutaneous tumors grew up to 200300 mm^3^, the subcutaneous CT26 murine tumor model was left untreated or intratumorally injected with SnSe NSs (4.5 mg/kg) or inSnSe NSs (4.5 mg/kg). After 24 h, tumors excised with 1 mL PBS from BALB/c mice were prepared into tumor homogenate by a tissue homogenizer. The lactate level of tumor homogenate was detected in the absorbance of supernatant at 530 nm on the microplate reader by the lactate assay kit. The BCA Protein Assay Kit (Beyotime Biotechnology, Jiancheng, Nanjing, China) was used to determine the protein concentration of the tumor homogenate to perform normalized data processing. The lactate level of the tumor homogenate was calculated using the formula: the absorbance/protein concentration.

### 3.11. In Vivo Cancer Treatment

The treatment efficiency of SnSe NSs or inSnSe NSs was evaluated in a model of CT26luc tumor bearing BALB/c mice. A total of 1 × 10^6^ CT26luc cells/50 μL PBS were implanted into the depilated BALB/c mice to establish a CT26 subcutaneous murine tumor model on day 0. When the tumor reached approximately 100 mm^3^, mice were divided into three groups (control, SnSe NSs, and inSnSe NSs) and were treated with 90 µg (dose of 4.5 mg/kg) of SnSe NSs or inSnSe NSs intratumorally every two days. To study the therapeutic effect of SnSe NSs in other tumor models, 1 × 10^6^ tumor cells/50 μL PBS (B16, 4T1) were injected subcutaneously into the mice to establish B16 and 4T1 subcutaneous murine tumor models. Subcutaneous murine tumor models were left untreated or intratumorally injected with SnSe NSs (dose of 4.5 mg/kg) every two days. To examine the therapeutic effect of SnSe NSs or SnSe NSs, tumor sizes were observed every other day, and tumor volume was calculated using the following formula: length × width × width/2. 

### 3.12. In Vivo Immune Activation of SnSe NSs

Flow cytometry was used to evaluate the immune microenvironment after SnSe NS treatment. Fluorochromeconjugated antibodies against mouse CD3, CD8, ki67, and IFNγ were purchased from BioLegend (San Diego, CA USA). After euthanizing the mice, tumor tissues excised from mice with sterile scissors were prepared into a tumor homogenate with 1 mL PBS using a tissue homogenizer. The single cell suspension was then filtered through 300 mesh nylon gauze. Cell surface staining was next performed by incubating cells with antibodies in flow cytometry buffer (3% bovine serum albumin in PBS) for 1.5 h. For the intracellular staining experimental procedure, cells were fixed and incubated with antibodies in an IntraPrep™ permeabilization reagent. Finally, the various stained cells were analysed using a BD Accuri™ C6 flow cytometer (Franklin Lakes, NJ, USA) [27].

### 3.13. Statistical Analysis

Unless otherwise stated, all data are presented as the mean ± SD. All statistical analyses were performed using Prism v8.0 (GraphPad, San Diego, CA, USA). Differences between two groups were assessed using a two-tailed unpaired Student’s *t*-test. In addition, one-way ANOVA was performed to compare more than two groups using the Tukey posttest. A *p*-value of 0.05 or less was considered significant. *p*-values are indicated as ** *p* < 0.05, *** *p* < 0.01, *** *p* < 0.001, and **** *p* < 0.0001.

## 4. Discussion and Conclusions

In recent years, targeting tumor lactate metabolism has become one of the hot spots in tumor metabolic therapy research. However, some therapeutic agents that target the lactate metabolic pathway may have the opposite effect on immune cells in the TME [28]. Compared with previously reported SnSe NS-based nanosystems and conventional lactate inhibitors targeting tumor metabolism [14], we found that SnSe NSs reacted with H+ of tumor lactate to effectively alleviate TME acidification and effectively neutralize acidic tumor pH. In addition, they can promote the improvement of function of immune cells in TME and facilitate the reversal of immunosuppressed TME.

We designed a nanozyme as an alternative to lactate dehydrogenase to inhibit tumor progression by depleting lactate to induce cell function activation. It has been shown that lactate drives the transcription of genes encoding VEGF and the arginine metabolizing enzyme arginase 1 (ARG1) in tumor-associated macrophages, driving them to polarize toward the M2 phenotype [13]. In vitro experiments, SnSe NSs react with lactate produced by intracellular metabolism to convert cellular lactate to pyruvate, thereby depleting lactate. Lactate depletion mediated by SnSe NSs leads to repolarization of TAMs to the M1 phenotype, resulting in restoration of tumor-killing activity of M1-type TAMs [14]. SnSe NSs act as lactate regulators and trigger intracellular signals that can affect cellular function and metabolic changes. However, whether SnSe NSs downregulate the transcription of these genes will be investigated in the future. In vivo experiments demonstrated that SnSe NSs can regulate lactate levels and neutralize tumor pH, as well as inhibit tumor growth. This was associated with a significant increase in the proportion and activation of CD8^+^ T cells. Our study showed that SnSe NSs provide a viable strategy for enhancing therapeutics targeting lactate metabolism and offer some insights into material design for tumor therapy.

## Data Availability

The data presented in this study are contained within the article.

## Supplementary Materials

The following supporting information can be downloaded at: https://www.mdpi.com/article/10.3390/molecules27238552/s1. Figure S1: Cytotoxicity of SnSe NSs with various concentrations in cancer cells. Figure S2: Confocal imaging of FITC-labelled SnSe NSs with different time points after incubation with RAW 264.7 cells for 24 h. Figure S3: Representative flow cytometry zebra plots and statistical analyses of CD4^+^ T cells after SnSe NSs treatments in a B16 tumor model.

## Sample Availability

Samples of the compounds are not available from the authors.
