# Supplementary material for: SnSe Nanosheets Mimic Lactate Dehydrogenase to Reverse Tumor Acid Microenvironment Metabolism for Enhancement of Tumor Therapy
Source: Molecules. 2022 Dec 5;27(23):8552. doi: 10.3390/molecules27238552 (PMC9738583; doi:10.3390/molecules27238552)
Supplement: Supplementary file 1 [file molecules-27-08552-s001.zip › molecules-2030190-supplementary.pdf]

Supplementary information for

SnSe Nanosheets Mimic Lactate Dehydrogenase to Reverse Tumor Acid Microenvironment Metabolism for Enhancement of Tumor Therapy

Heng Wang[1†], Beilei Wang[1†], Jie Jiang[2†], Yi Wu[1], Anning Song[1], Xiaoyu Wang[1], Chenlu Yao[1], Huaxing Dai[1], Jialu Xu[1], Yue Zhang[1], Qingle Ma[1], Fang Xu[1], Ruibin Li[2], Chao Wang[1*]

[1] Institute of Functional Nano & Soft Materials (FUNSOM), Soochow University, Suzhou, 215123, China

[2] State Key Laboratory of Radiation Medicine and Protection, School for Radiological and Interdisciplinary Sciences (RAD-X), Collaborative Innovation Center of Radiation Medicine of Jiangsu Higher Education Institutions, Soochow University, Suzhou 215123, China

* Corresponding author: Chao Wang: cwang@suda.edu.cn

†These authors contributed equally to this work .

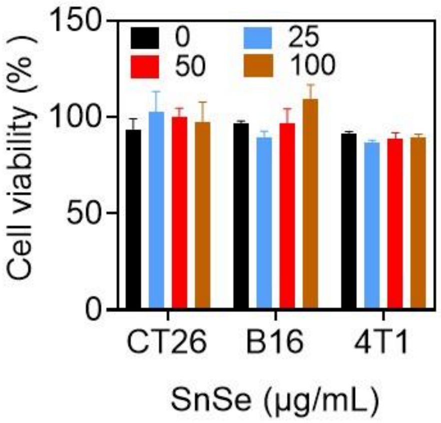

Figure S1. Cytotoxicity of SnSe NSs with various concentrations to cancer cells. Data are means ± SD.

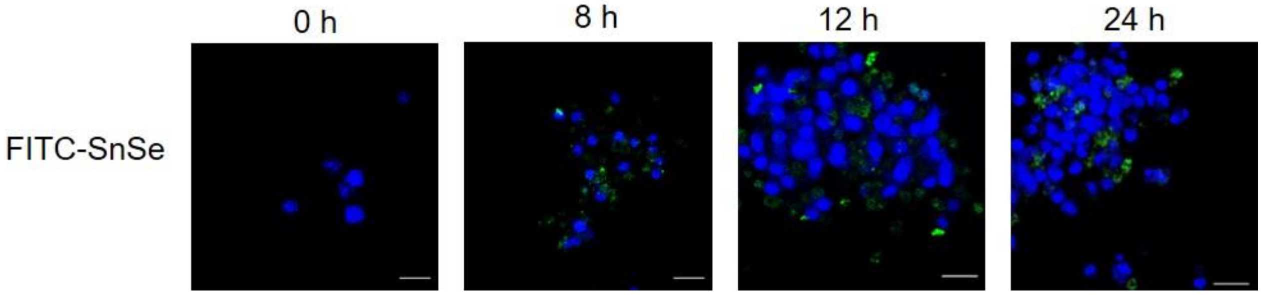

Figure S2. Confocal imaging of FITC-labelled SnSe NSs with different time points after incubation with RAW 264.7 cells for 24 h (blue: DAPI; green: FITC; Scale bar = 20 μm).

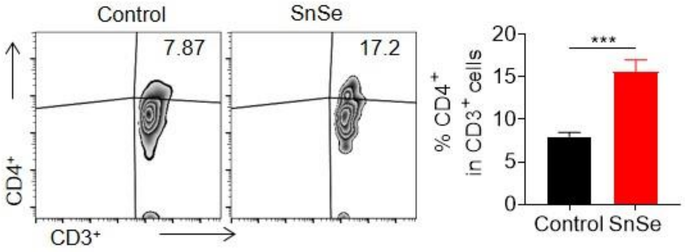

Figure S3. Representative flow cytometry zebra plots and statistical analyses of $CD4^+$ T cells in B16 tumor model after SnSe NSs treatments. Data are means ± SD. Statistical significance between different groups was obtained by Student's t tests (two-tailed). *P*-value: *** $P < 0.001$. n.s., no significance. a.u., arbitrary units.
